# 改进的QuEChERS结合超高效液相色谱-串联质谱法检测饲料中的环匹阿尼酸

**DOI:** 10.3724/SP.J.1123.2023.10030

**Published:** 2024-05-08

**Authors:** Maomin PENG, Xiaobing YU, Lin CHEN, Qingsong XIONG, Li LIU, Dan ZHENG, Hong XIA, Qiongwei YU, Xitian PENG

**Affiliations:** 1.湖北省农业科学院农业质量标准与检测技术研究所, 农产品营养品质与安全湖北省重点实验室, 湖北 武汉 430064; 1. Institute of Agricultural Quality Standards and Testing Technology Research, Hubei Academy of Agricultural Science, Hubei Key Laboratory of Nutritional Quality and Safety of Agro-products, Wuhan 430064, China; 2.荆州市农业农村局, 湖北 荆州 434020; 2. Jingzhou Agriculture and Rural Bureau, Jingzhou 434020, China; 3.湖北省食品质量安全监督检验研究院, 湖北 武汉 430075; 3. Hubei Provincial Institute for Food Supervision and Test, Wuhan 430075, China; 4.武汉大学化学与分子科学学院, 湖北 武汉 430072; 4. College of Chemistry and Molecular Sciences, Wuhan University, Wuhan 430072, China

**Keywords:** QuEChERS, 超高效液相色谱-串联质谱, 环匹阿尼酸, 饲料, QuEChERS, ultra performance liquid chromatography-tandem mass spectrometry (UPLC-MS/MS), cyclopiazonic acid (CPA), feeds

## Abstract

采用改进的QuEChERS前处理方法结合超高效液相色谱-串联质谱(UPLC-MS/MS)分离检测,建立了饲料中真菌毒素环匹阿尼酸(CPA)的快速分析新方法。详细优化了UPLC-MS/MS分离检测条件和影响CPA回收率的QuEChERS条件,优化后的分析方法如下:1.0 g饲料加入2 mL水和4 mL 0.5%乙酸乙腈溶液进行提取,再加入提取盐包(0.4 g氯化钠和1.6 g无水硫酸镁),离心分层后,取1 mL乙腈提取液,加入150 mg无水硫酸镁和50 mg C_18_吸附净化,上清液进行UPLC-MS/MS分离检测;采用Waters HSS T3柱(100 mm×2.1 mm, 1.8 μm),以含0.5%甲酸的2 mmol/L乙酸铵水溶液和乙腈作为流动相,梯度洗脱,电喷雾正离子模式下以多反应监测(MRM)扫描模式定量。CPA在2~200 ng/mL的范围内具有良好的线性,相关系数良好(*r*=0.9995),方法的检出限和定量限分别为0.6和2 μg/kg,饲料中CPA在10、100、500 μg/kg 3个加标水平下的回收率为70.1%~78.5%,日内RSD为4.1%~5.8%,日间RSD为6.1%~7.2%。将本方法应用于实际饲料样品中CPA的分析,取得了很好的效果。本研究将改进的QuEChERS方法应用于饲料中CPA的提取净化,为饲料中CPA的风险监测、评估和限量标准制定提供了一种有效的检测技术。

## Abstract (en)

Mycotoxins are toxic secondary metabolites produced by fungal species that can cause acute, subacute, and chronic toxicity in humans and animals. Thus, these toxins pose a significant threat to health and safety. Owing to the lack of effective antimold measures in the agricultural industry, feed ingredients such as corn, peanuts, wheat, barley, millet, nuts, oily feed, forage, and their byproducts are prone to mold and mycotoxin contamination, which can affect animal production, product quality, and safety. Cyclopiazonic acid (CPA), which is mainly biosynthesized from mevalonate, tryptophan, and diacetate units, is a myotoxic secondary metabolite produced by *Penicillium* and *Aspergillus* fungi. CPA is widely present as a copollutant with aflatoxins in various crops. Compared with some common mycotoxins such as aflatoxins, fumonisins, ochratoxins, zearalenones, and their metabolites, CPA has not been well investigated. In the United States, a survey showed that 51% of corn and 90% of peanut samples contained CPA, with a maximum level of 2.9 mg/kg. In Europe, CPA was found in *Penicillium*-contaminated cheeses as high as 4.0 mg/kg. Some studies have shown that CPA can cause irreversible damage to organs such as the liver and spleen in mice. Therefore, the establishment of a rapid and efficient analytical method for CPA is of great significance for the risk assessment of CPA in feeds, the development of standard limits, and the protection of feed product quality and safety. The QuEChERS method, a sample pretreatment method that is fast, simple, cheap, effective, and safe, is widely used in the analysis of pesticide residues in food.

In this study, a modified QuEChERS method combined with ultra performance liquid chromatography-tandem mass spectrometry (UPLC-MS/MS) was used to determine CPA levels in feeds. The chromatographic separation and MS detection of CPA as well as the key factors affecting the extraction efficiency of CPA, including the type of extraction solvent, type of inorganic salt, and type and dosage of adsorbent, were optimized in detail. During the optimization of the chromatographic-separation step, the acid and salt concentrations of the mobile phase affected the separation and detection of CPA. During the optimization of the QuEChERS method, the addition of a certain amount of acetic acid improved the extraction efficiency of CPA because of its acidic nature; in addition, GCB and PSA significantly adsorbed CPA from the feed extract. Under optimal conditions, the CPA in the feed sample (1.0 g) was extracted with 2 mL of water and 4 mL of acetonitrile (ACN) containing 0.5% acetic acid. After salting out with 0.4 g of NaCl and 1.6 g of MgSO_4_, 1 mL of the ACN supernatant was purified by dispersive solid-phase extraction using 150 mg of MgSO_4_ and 50 mg of C_18_ and analyzed by UPLC-MS/MS. The sample was separated on a Waters HSS T3 column (100 mm×2.1 mm, 1.8 μm) using 2 mmol/L ammonium acetate aqueous solution with 0.5% formic acid and ACN as the mobile phases and then analyzed by positive electrospray ionization in multiple reaction monitoring mode. CPA exhibited good linearity in the range of 2-200 ng/mL, with a high correlation coefficient (*r*=0.9995). The limits of detection and quantification of CPA, which were calculated as 3 and 10 times the signal-to-noise ratio, respectively, were 0.6 and 2.0 μg/kg, respectively. The average recoveries in feed samples spiked with 10, 100, and 500 μg/kg CPA ranged from 70.1% to 78.5%, with an intra-day precision of less than 5.8% and an inter-day precision of less than 7.2%, indicating the good accuracy and precision of the proposed method. Finally, the modified QuEChERS-UPLC-MS/MS method was applied to the analysis of CPA in 10 feed samples obtained from Wuhan market. The analysis results indicated that the developed method has good applicability for CPA analysis in feed samples. In summary, an improved QuEChERS method was applied to the extraction and purification of CPA from feeds for the first time; this method provides a suitable analytical method for the risk monitoring, assessment, and standard-limit setting of CPA in feed samples.

真菌毒素是由丝状真菌产生的、具有毒性的次级代谢产物,可以使人和动物出现急性、亚急性和慢性中毒等症状,对人类和动物健康安全产生了较大的影响^[1⇓-3]^。据统计,在全世界每年浪费的农产品中,被真菌毒素污染的农产品占到了总量的1/5,损失金额高达数百亿美元^[4]^。饲料原料如玉米、花生、小麦、大麦、小米、坚果和油性饲料、饲草及其副产品由于缺少有效防霉措施,容易被真菌感染后霉变,产生有毒的次生代谢产物真菌毒素,从而影响动物生产以及产品质量安全^[5,6]^。因此,我国对于饲料原料和全价饲料中多种真菌毒素如黄曲霉毒素(AFB1)、脱氧雪腐镰刀菌素(DON)和玉米赤霉烯酮(ZEN)设立了限量标准,分别为10~20、1 000~5 000和100~250 μg/kg,以保障饲料质量安全。

环匹阿尼酸(CPA)是目前关注较少的一种真菌毒素(结构式见图1),是青霉和曲霉属真菌产生的具有肌肉毒性的次级代谢产物,并作为黄曲霉毒素的共同污染物广泛存在于各种作物产品中^[7]^。在美国,一项调查表明51%的玉米和90%的花生样品中含有CPA,最高含量达到了2.9 mg/kg;在欧洲的一项研究表明,CPA在被青霉菌污染的奶酪中的含量高达4.0 mg/kg^[5]^。研究表明,CPA能对小鼠的肝脏、脾脏等器官造成不可逆转的伤害,还会对它们的神经造成严重损害,同时也会对人体产生细胞毒性、血液疾病等危害^[5,8]^。因此,建立饲料中CPA快速高效的分析方法,对于饲料中CPA的安全风险评估、限量标准的制定,以及保障饲料产品质量安全具有重要的意义。目前,文献已报道了多种CPA分析方法,如酶联免疫法^[7,9]^、毛细管电泳法^[10]^、液相色谱法^[11⇓-13]^以及液相色谱-质谱法^[5,14⇓⇓⇓-18]^。其中,液相色谱-质谱法应用最为广泛,与其他方法相比,液相色谱-质谱法具有更加准确的定性能力以及较高的灵敏度、选择性和抗干扰能力^[19]^。

**图1:**
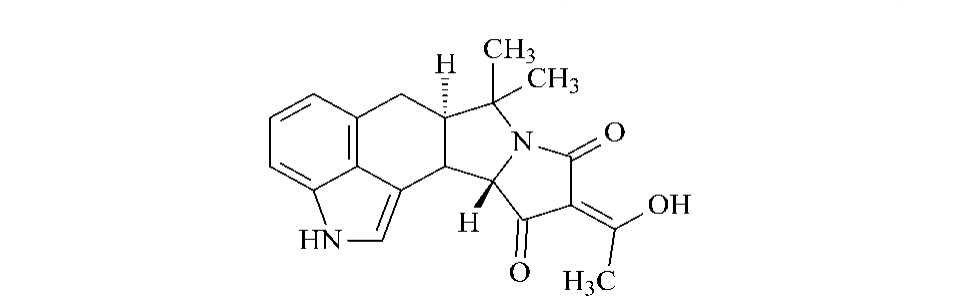
CPA的结构式

饲料样品基质非常复杂,脂类物质含量高,CPA含量很低,要检测其中的CPA含量,需要通过一定的样品前处理方法来对其进行提取净化,减少样品基质对色谱、质谱分析结果和仪器使用寿命的影响。QuEChERS法是目前在食品中农药残留分析领域得到广泛应用的一种新型样品前处理方法。传统的QuEChERS采用乙腈(ACN)提取,添加无机盐液液分层后,采用分散固相萃取净化,具有快速、简单、便宜、有效和安全等特点^[20,21]^。Peromingo等^[14]^采用QuEChERS结合高效液相色谱-串联质谱建立了腌制火腿中CPA高效灵敏的分析方法,目前还未见QuEChERS用作饲料中CPA含量分析的报道。本文在保留传统QuEChERS原有步骤的基础上,针对特定的分析物CPA,改进前处理过程中的关键步骤,并结合超高效液相色谱-串联质谱法(UPLC-MS/MS)构建了快速分析饲料中CPA的方法,在实际样品分析中取得了较好的效果。

## 1 实验部分

### 1.1 仪器设备

LC 40Dx3超高效液相色谱仪(日本Shimadzu公司),Triple Quad 5500^+^质谱仪(美国Sciex公司);数据采集由Analyst^@^工作站完成,数据处理由MultiQuant分析软件完成;Milli-Q A-10超纯水仪(美国Millipore公司); NR2002E型电子天平(上海梅特勒-托利多仪器有限公司); TDL-5C台式大容量离心机(上海安亭科学仪器厂)。

### 1.2 试剂及材料

无水硫酸镁、氯化钠、醋酸钠、柠檬酸钠和乙酸均为分析纯,购自国药集团化学试剂公司;乙腈、甲醇、甲酸和乙酸铵均为色谱纯,购自上海安谱实验科技有限公司;CPA标准品(纯度>98%)购自上海毕得医药科技有限公司;*N*-丙基乙二胺(PSA)、十八烷基键合硅胶(C_18_)和石墨化炭黑(GCB)购自天津博纳艾杰尔科技有限公司;10个饲料样品购自武汉本地市场。

### 1.3 标准溶液的配制

标准储备液:称取5.0 mg CPA标准品于25 mL棕色容量瓶中,加入乙腈溶解定容,得到200 mg/L的标准储备液,-20 ℃避光保存。再吸取200 mg/L的CPA标准储备液0.1 mL,用乙腈定容至10 mL,得到2 mg/L的标准溶液,4 ℃冰箱保存备用。

基质标准工作液:将空白饲料样品经过前处理得到的溶液作为稀释溶剂。吸取适量2 mg/L的标准溶液,用稀释溶剂配制质量浓度为2、5、10、20、50、100和200 ng/mL的基质标准溶液。

### 1.4 样品前处理

准确称取1.0 g饲料样品于15 mL离心管中,加入2 mL去离子水,涡旋振荡1 min,使饲料样品与水充分混合,随后加入4 mL含0.5%乙酸的乙腈溶液,涡旋振荡1 min。向离心管中加入提取盐包(0.4 g氯化钠和1.6 g无水硫酸镁),涡旋振荡1 min后放入离心机中以4200 r/min离心5 min,取1 mL上清液至净化管(含150 mg无水硫酸镁和50 mg C_18_)中,涡旋振荡1 min后,以4200 r/min离心5 min,上清液过0.22 μm尼龙膜后,待分析。

### 1.5 分析条件

采用沃特世科技(上海)有限公司ACQUITY UPLC^®^ HSS T3色谱柱(100 mm×2.1 mm, 1.8 μm);流动相A:含有0.5%甲酸的2 mmol/L乙酸铵水溶液,流动相B:乙腈;流速:0.3 mL/min。梯度洗脱程序:0~0.6 min, 30%B; 0.6~3.0 min, 30%B~95%B; 3.0~5.8 min, 95%B; 5.80~5.85 min, 95%B~30%B; 5.85~7.40 min, 30%B。柱温:40 ℃;进样体积:5 μL。

离子源:电喷雾电离(ESI)源,正离子模式;扫描方式:多反应监测模式(MRM);离子化电压(IS): 5500 V;离子源温度:500 ℃;气帘气压力(CUR): 241.5 kPa(35 psi);喷雾气压力(GS1): 276 kPa(40 psi);辅助加热气压力(GS2): 276 kPa(40 psi);碰撞气压力:62.1 kPa(9 psi)。其他质谱参数,包括母离子、子离子、去簇电压(DP)及碰撞能量(CE)见表1。

**表1:** CPA的保留时间和质谱参数

Compound	Retention time/min	Parent ion (m/z)	Daughter ion (m/z)	DP/ V	CE/ eV
CPA	3.85	337.0	196.0^*^	130	31
		337.0	182.0	130	25

* Quantitative ion; DP: declustering potential; CE: collision energy.

## 2 结果与讨论

### 2.1 质谱条件的优化

将200 ng/mL的CPA标准溶液直接注射到质谱仪中,用于优化质谱条件。在负离子模式下,Q1全扫描发现[M-H]^-^ (*m/z* 335.0)峰响应高,随后通过子离子扫描,发现母离子破碎后会得到*m/z*为154.0和140.0的子离子,进一步优化质谱条件,得到对应的去簇电压和碰撞能量。然而,通过色谱进样后发现CPA质谱响应较低,这可能是由于水相中甲酸含量较高,在负离子模式下CPA的电离被抑制。在正离子模式下,扫描得到[M+H]^+^(*m/z* 337.0)的母离子,进一步得到*m/z*为196.0和182.0的子离子及其对应的去簇电压和碰撞能量。通过色谱进样后发现CPA质谱响应可以满足分析要求,选择其中响应较高的337.0/196.0为定量离子对,337.0/182.0为定性离子对。

### 2.2 色谱条件的优化

使用20 ng/mL的CPA标准溶液进样优化色谱分离条件,实验考察了3种不同类型的色谱柱(ACQUITY UPLC^®^ BEH C_18_ (50 mm×2.1 mm, 1.7 μm)、ACQUITY UPLC^®^ BEH Shield RP18 (100 mm×2.1 mm, 1.7 μm)、ACQUITY UPLC^®^ HSS T3(100 mm×2.1 mm, 1.8 μm))的分离性能,比较其保留时间和峰形。结果显示,CPA在HSS T3柱上的响应大于另外两种色谱柱,且保留时间更长,有利于目标物与基质成分分离,因此选其作为分离分析色谱柱。

进一步对流动相条件进行了优化。当有机相为甲醇或者乙腈时,CPA的峰形和峰面积变化不明显,因为乙腈洗脱能力强,系统压力小,我们选用乙腈作为有机相。当水相中的甲酸体积分数较低时,色谱峰拖尾严重,随着甲酸体积分数的增加,色谱峰形逐渐改善,当甲酸体积分数达到0.5%时,色谱峰形较好。实验考察了水相中不同浓度乙酸铵和不同体积分数甲酸对CPA峰面积的影响,结果见图2。在各种乙酸铵浓度下,含0.5%甲酸的水相均能得到更大的峰面积;而随着乙酸铵浓度的增加,峰面积先增加后减小,乙酸铵为2 mmol/L时,峰面积达到最高。因此,本研究采用含0.5%甲酸的2 mmol/L乙酸铵溶液作为水相。

**图2:**
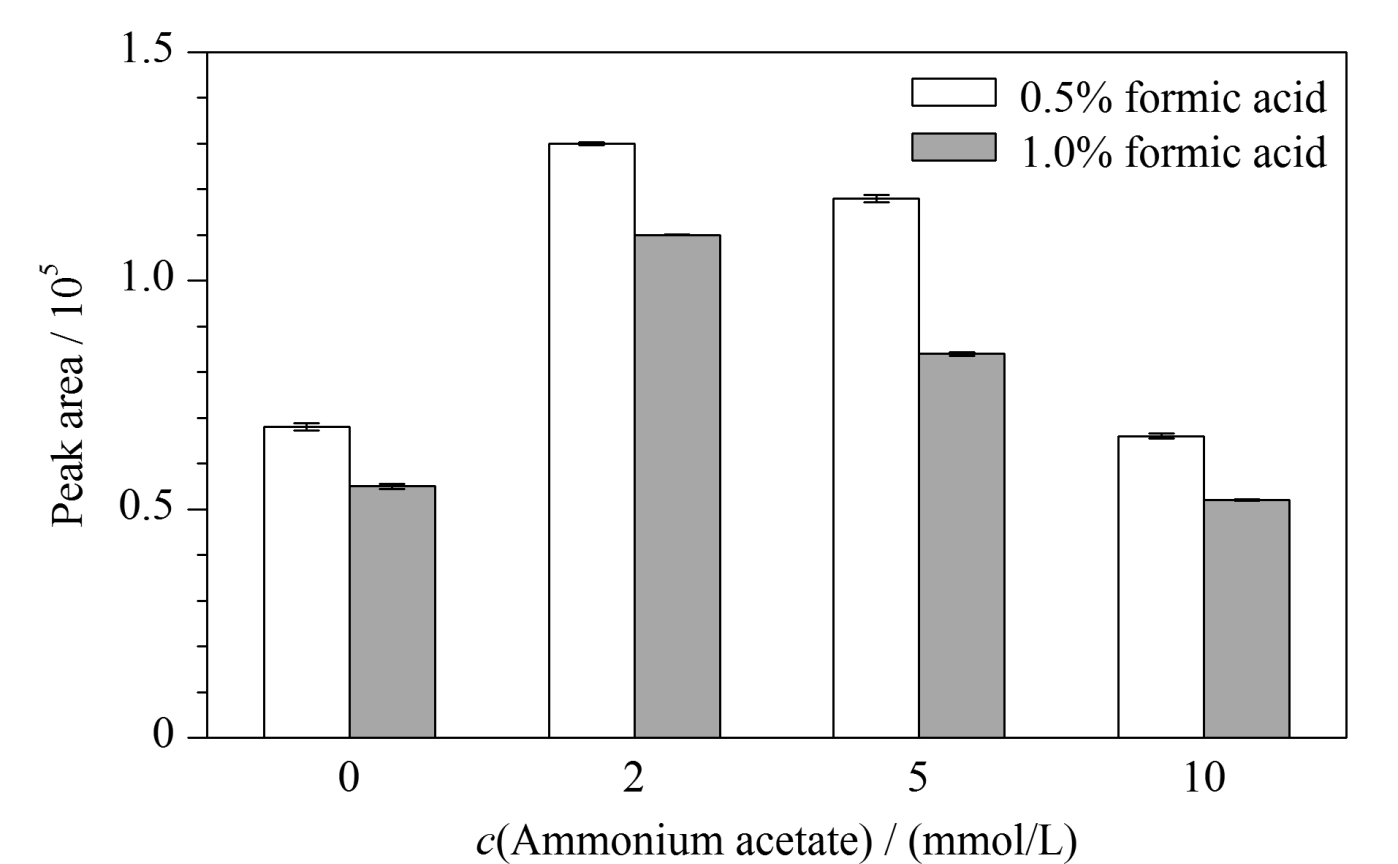
不同水相条件下CPA的峰面积(*n*=3)

在优化条件下CPA标准溶液和加标样品的MRM色谱图见图3。

**图3:**
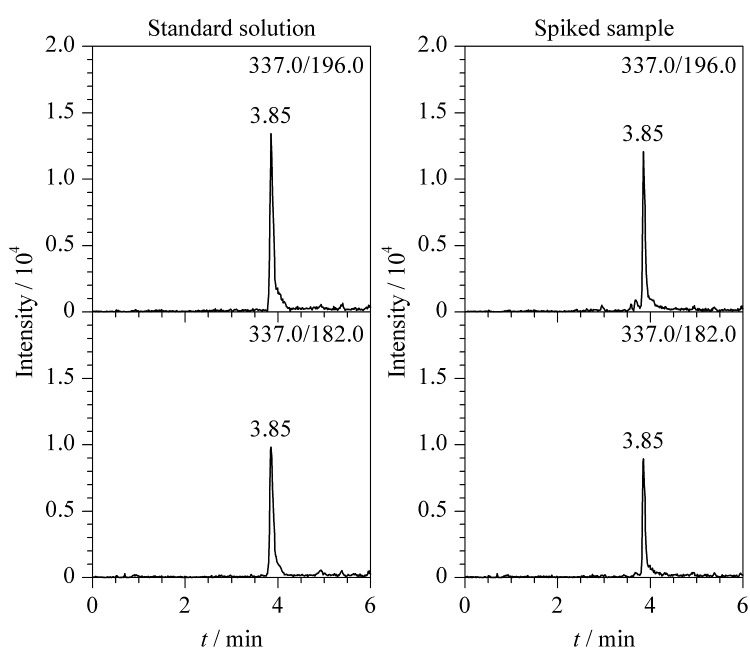
CPA标准溶液和加标样品的MRM色谱图

### 2.3 改进的QuEChERS条件的优化

为了提高CPA的提取效率,以CPA加标水平为20 μg/kg的空白饲料作为试验样品,考察了不同的QuEChERS条件对CPA提取效率的影响。

#### 2.3.1 提取溶剂的优化

CPA是一种酸性物质,提取溶剂的酸度对其回收率有较大影响,用含有不同体积分数乙酸的乙腈溶液对加标饲料样品进行提取,分别经过净化(150 mg无水硫酸镁和50 mg C_18_)和不净化处理,检测样品中CPA的含量,结果见图4a。样品净化后,CPA回收率比无净化过程更高,这表明通过净化,降低了基质对目标物的影响;同时,CPA回收率在乙酸体积分数为0.5%时达到最高,此时提取效果最好。传统的QuEChERS方法采用纯乙腈作为提取溶剂,本研究中目标物CPA的p*K*_a_为2.97^[22]^,乙酸的加入可以有效抑制CPA的电离,增加其在有机相中的分配比例,从而提高CPA的回收率。同时,CPA结构不稳定,乙酸体积分数过高可能导致其稳定性降低。因此选择含0.5%乙酸的乙腈溶液为提取溶剂,且需要净化过程。

**图4:**
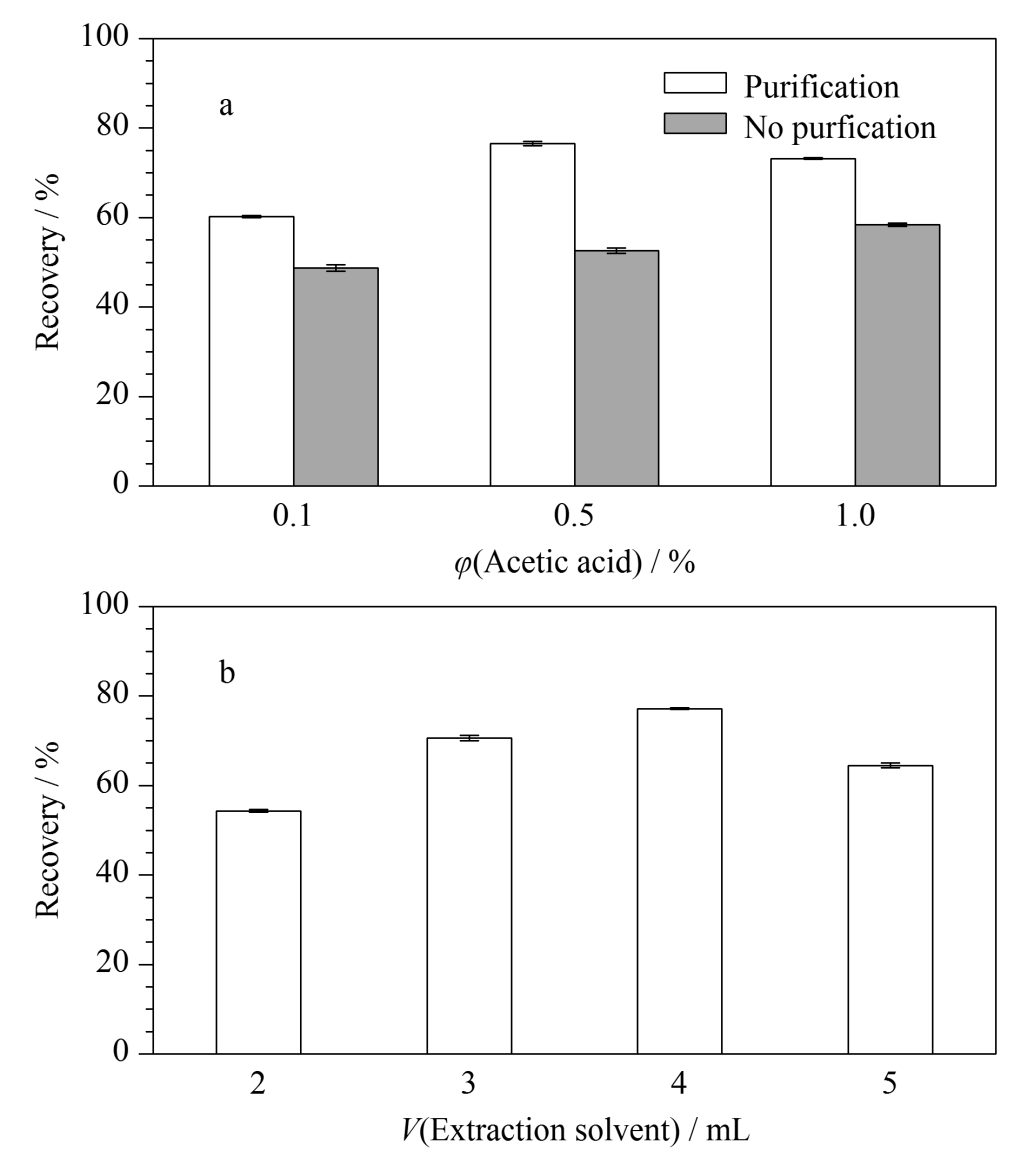
提取溶剂的(a)组成和(b)体积对CPA回收率的影响(*n*=3)

在加标饲料样品中分别加入2、3、4、5 mL的提取溶剂(含0.5%乙酸的乙腈溶液),前处理后进样分析,结果见图4b。随着提取溶剂体积的不断增加,CPA的回收率先增加后减小,在提取溶剂体积为4 mL时,回收率最高,达到80%左右,因此将提取溶剂的体积定为4 mL。相比传统的QuEChERS方法中样品与提取溶剂的比例为1∶1,本研究增加了提取溶剂的体积,一方面提高了样品的提取回收率,另一方面也通过样品的稀释降低了基质的干扰。

#### 2.3.2 无机盐的优化

QuEChERS的提取过程中需要添加无机盐,以促使有机相和水相分层,同时盐包成分和提取溶剂相互作用,形成不同的缓冲体系,有利于提取体系维持在一定的pH范围内,保证应用于不同样品时提取效率的稳定性。本研究考察了3种常见的无机盐组成对CPA提取效率的影响,结果见图5。当使用无机盐组成为0.4 g氯化钠和1.6 g无水硫酸镁时,CPA的回收率最大(79%),而使用其他两种无机盐组成(0.4 g醋酸钠和1.6 g无水硫酸镁、0.4 g柠檬酸钠和1.6 g无水硫酸镁)时回收率低于60%,因此将0.4 g氯化钠和1.6 g无水硫酸镁作为CPA提取时的无机盐。

**图5:**
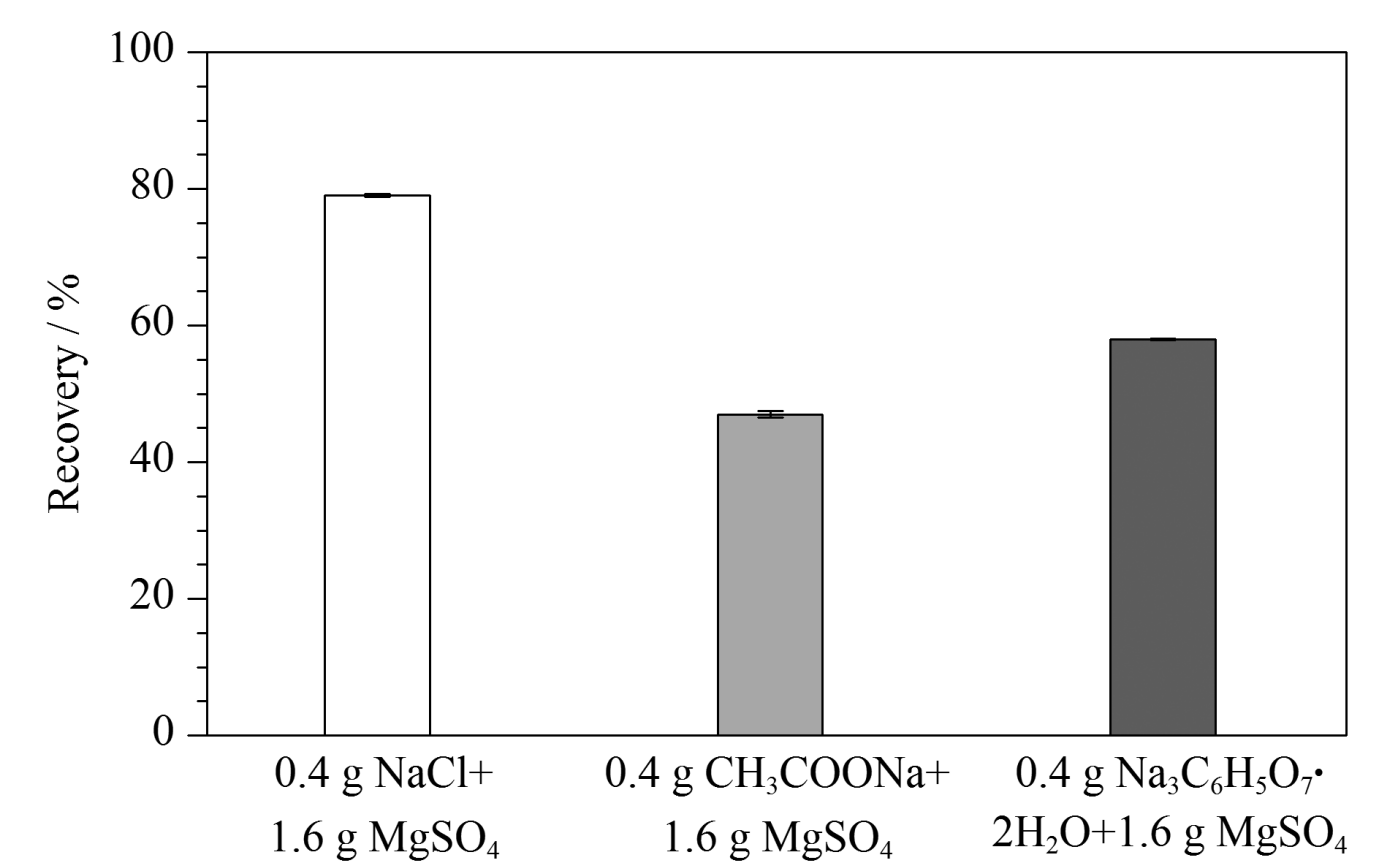
提取盐包成分对CPA回收率的影响(*n*=3)

#### 2.3.3 吸附剂的选择

2003年,Anastassiades等^[21]^报道了QuEChERS方法应用于果蔬中农药残留的分析,使用的吸附剂为PSA,其通过氨基与基质之间的极性相互作用吸附和消除样品基质中的糖类、色素以及脂肪酸。随后,很多研究者针对不同的基质和目标物对吸附剂的类型进行了改进,其中C_18_和GCB是目前应用较多的吸附剂。C_18_对脂类物质具有较好的吸附作用,而GCB对基质有强烈的吸附作用,但同时对非极性农药和具有平面结构的物质也有一定的吸附作用。吸附剂的选择和用量是影响净化效果的关键,既要能够吸附样品中的基质,减少基质对目标物的影响,又要避免吸附剂吸附目标物,影响回收率。本研究考察了不同的吸附剂组合对CPA回收率的影响,结果见图6a。吸附剂为150 mg无水硫酸镁和50 mg C_18_时的回收率最高,接近80%,而加入PSA或GCB时,都会显著降低回收率。原因可能在于,PSA中的氨基部分会与酸性的CPA通过离子相互作用结合,同时CPA结构中带有苯环,具有平面结构,也容易被GCB吸附,造成目标物回收率的降低。

**图6:**
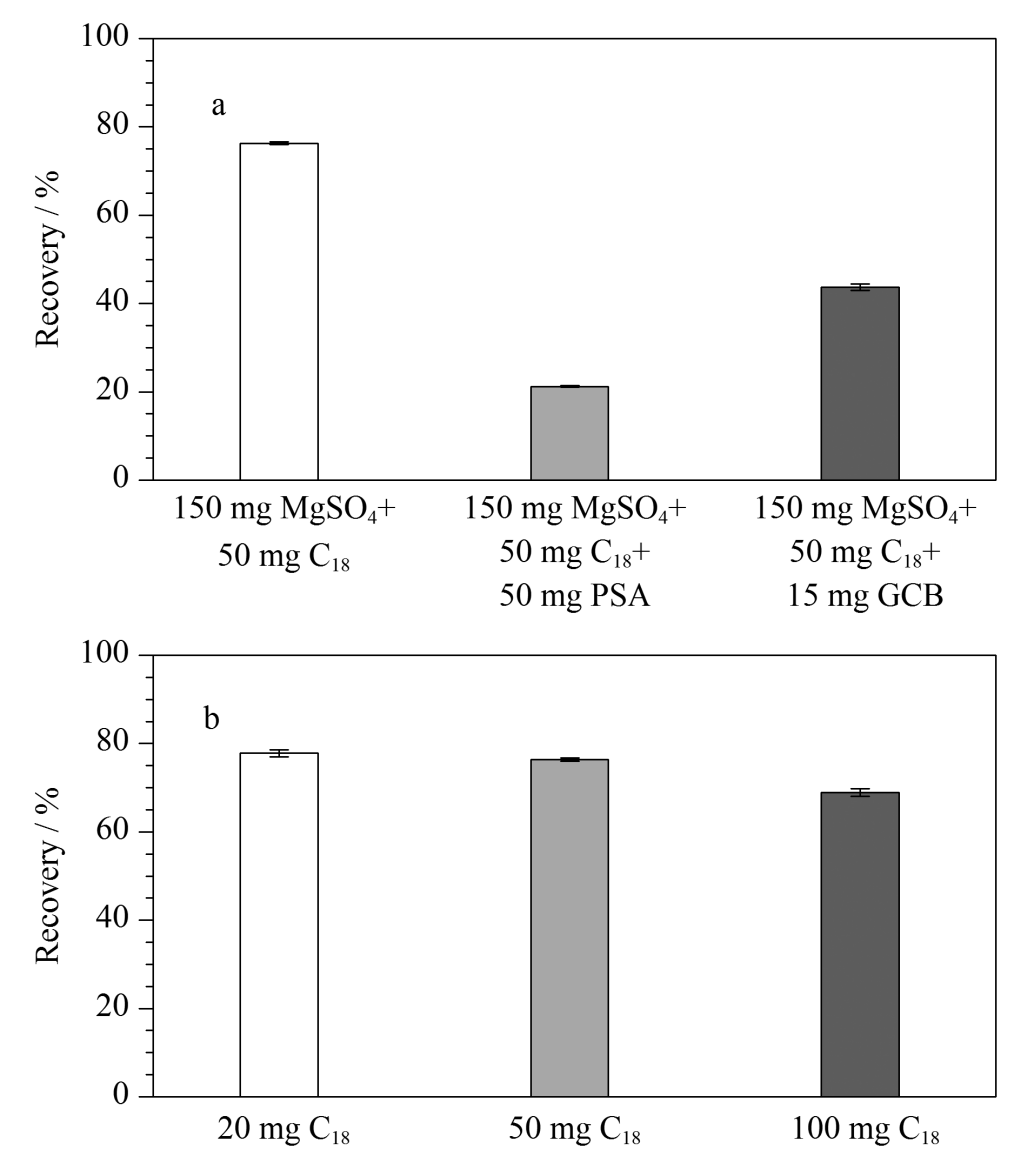
不同(a)吸附剂和(b)C_18_含量对CPA回收率的影响(*n*=3)

实验继续考察了C_18_的最佳使用量,在150 mg无水硫酸镁的基础上分别添加不同质量的C_18_,结果见图6b。随着净化剂中C_18_质量的增加,CPA的回收率逐渐减小,但差别较小。综合考虑回收率和净化效果,最终选择吸附剂中C_18_用量为50 mg。

### 2.4 方法学考察

#### 2.4.1 基质效应和线性方程

在质谱分析中,样品溶液中的基质会干扰目标物在离子源中的电离行为,影响目标物的离子化效率,从而影响目标物的响应,这被称为基质效应(ME)。衡量基质效应的方式有多种,其中一种是以(基质匹配标准曲线斜率/溶剂标准曲线斜率-1)×100%)来表示。一般来说,|ME|小于20%为弱基质效应,在20%~50%范围内为中等基质效应,大于50%为强基质效应。用纯溶剂和空白样品经前处理后得到的溶液作为稀释溶剂,分别配制质量浓度为2、5、10、20、50、100、200 ng/mL的CPA系列标准溶液,按照给定的色谱-质谱条件进行UPLC-MS/MS检测,以CPA的质量浓度为横坐标(*X*, ng/mL),相应的定量离子峰面积为纵坐标(*Y*),绘制溶剂标准溶液和基质匹配标准溶液的校正曲线,得到二者的线性方程分别为*Y*=4.43×10^3^*X*+2.81×10^2^(*r*=0.9920)和*Y*=3.15×10^3^*X*+2.34×10^2^(*r*=0.9995),计算出基质效应为-28.8%,表明经本方法前处理后还存在中等基质效应。因此,本研究采用基质匹配标准溶液来消除或者减弱基质效应的影响。同时,以3倍和10倍信噪比计算CPA的检出限和定量限,结果分别为0.6 μg/kg和2.0 μg/kg。

#### 2.4.2 准确度和精密度

通过加标样品的回收率来评价方法的准确度,通过日内、日间回收率的相对标准偏差(RSD)来评价方法的精密度。在空白饲料样品中添加低(10 μg/kg)、中(100 μg/kg)、高(500 μg/kg)3个水平的CPA,每个添加水平设置6个平行样品,连续测定3天,结果见表2。结果表明,CPA在不同加标水平下的加标回收率为70.1%~78.5%,日内RSD范围为4.1%~5.8%,日间RSD范围为6.1%~7.2%,表明方法的准确度和精密度较好,能够满足检测工作的需要。

**表2:** CPA在饲料样品中的加标回收率和RSD

Compound	Spiked/ (μg/kg)	Intra-day (n=6)			Inter-day (n=3)	
		Recovery/ %	RSD/ %		Recovery/ %	RSD/ %
CPA	10	74.2	5.8		78.5	7.2
	100	70.9	4.7		72.5	6.8
	500	70.1	4.1		70.6	6.1

#### 2.4.3 实际样品的检测

购买了10种不同品牌、不同性状的饲料样品进行检测,其中5个样品检出CPA,含量范围为10.8~57.6 μg/kg,表明目前饲料中CPA的污染情况较为普遍,需引起重视,有必要开展饲料中CPA污染的风险评估。

## 3 结论

本文将改进的QuEChERS方法结合UPLC-MS/MS用于饲料中CPA的检测。对仪器检测条件和影响CPA回收率的QuEChERs条件进行了详细的优化。相比于传统的QuEChERs方法,本研究对提取溶剂的种类和比例、吸附溶剂的类型进行了改进,提高了CPA的回收率。结果表明,本研究建立的方法具有操作简单、灵敏度高、准确度好等优点。对10个饲料样品进行分析,其中在5个样品中检出CPA,表明本方法在饲料CPA的分析中具有很好的适用性,为饲料中CPA的风险监测、评估和标准限量制订提供了很好的分析方法支撑。
